# High-resolution Staphylococcus profiling reveals intra-species diversity in a single skin niche

**DOI:** 10.1099/mgen.0.001531

**Published:** 2025-10-08

**Authors:** Reyme Herman, Daniel T. West, Sean Meaden, Imran Khan, Robert Cornmell, Joanne Hunt, Michelle Rudden, Holly N. Wilkinson, Matthew J. Hardman, Anthony J. Wilkinson, Barry Murphy, Gavin H. Thomas

**Affiliations:** 1 Department of Biology, University of York, Wentworth Way, York, YO10 5DD, UK; 2 Unilever Research & Development, Port Sunlight Laboratory, Quarry Road East, Bebington, Wirral, Merseyside, CH63 3JW, UK; 3 Centre for Biomedicine, Hull York Medical School, University of Hull, Hull, HU6 7RX, UK; 4 York Structural Biology Laboratory, Department of Chemistry, University of York, Wentworth Way, York, YO10 5DD, UK

**Keywords:** *Staphylococcus*, skin microbiome, sequencing, comparative genomics

## Abstract

The skin microbiome is dominated by a few key genera, among which *Staphylococcus* is one of the most well characterized. Recent studies have examined the roles of various *Staphylococcus* species such as *Staphylococcus epidermidis* and *Staphylococcus hominis* within broader skin microbial communities. However, these investigations often rely on isolates from multiple individuals and hence limit their ability to capture intra-community interactions. In this study, we focused on the axillary microbiome of a single healthy individual to characterize the genetic and functional diversity of resident *Staphylococcus* isolates. Using a low-cost, high-throughput DNA extraction and long-read whole-genome sequencing pipeline, we generated complete genomes for 93 isolates spanning 7 genetically distinct lineages across 3 major skin species. These comprised one dominant and three additional lineages of *S. epidermidis*, two of *S. hominis* and one of *Staphylococcus capitis*. Functional and metabolic analyses revealed species- and strain-specific features, suggesting potential metabolic cross-feeding and specialization within this community, including within strains of *S. epidermidis*. These findings highlight the metabolic complexity and potential interdependence of staphylococci inhabiting a single skin site and the need for strain-level resolution of the community. The strains form part of the York Skin Microbiome (YSM) collection, a growing open biobank of genetically diverse skin isolates from matched individuals.

**Impact Statement**

Understanding how closely related microbial strains co-exist and interact within the same human body site is critical for deciphering the ecological and functional dynamics of the skin microbiome. This research pushes the boundaries of microbiome studies by employing a high-throughput long-read genome sequencing strategy on a single skin niche, revealing that strain-level genomic and functional diversity among coagulase-negative *Staphylococcus* is not only present but likely ecologically relevant. The discovery of discrete biosynthetic capacities, nutrient dependencies and antimicrobial resistance traits, even among isolates of the same species, suggests a division of labour and potential metabolic cross-feeding within the community. These findings shift our understanding of skin microbiota from being merely species-rich to being structured by fine-scale functional variation.

Beyond its biological insights, this work provides a scalable, low-cost sequencing and bioinformatic framework for microbiome studies. This work forms part of the York Skin Microbiome (YSM) collection: a curated, genomically resolved resource for experimental and translational research. By anchoring microbiome investigations at the strain level, this approach enables more precise interrogation of host–microbe and microbe–microbe interactions, informing therapeutic development. As public interest grows in microbiome-targeted interventions, these results underscore the importance of moving beyond species averages to capture the true complexity within our microbial ecosystems.

## Data Availability

Genomes of the seven dereplicated genomes were deposited to the GenBank repository within the BioProject PRJNA1191350. They are also publicly available for full analysis and download in the York Skin Microbiome Project on MORF (https://morf-db.org/projects/Public/MORF000083) where the genomes and orthologues across strains of the same species can be freely analysed.

## Introduction

Human skin is home to a wide range of micro-organisms that have evolved with their host over many millions of years [1,3]. Naturally, these organisms interact with each other and the host in a symbiotic relationship to establish a favourable environment which protects the host while allowing for these micro-organisms to thrive in generally nutrient-limiting conditions [4,5]. The multiple skin sites of the human body have some characteristic differences, such as being oily/sebaceous (e.g. face), dry/sebaceous poor (e.g. volar forearm) or moist (e.g. underarm/axilla), which then dictates the abundance and the diversity of microbes in the microbiome. Multiple synergistic and antagonistic relationships exist in these niches which helps maintain a healthy environment for the host [4]. The genus *Staphylococcus* is one of the most abundant genera on human skin and can be found in multiple skin environments. While some species within this genus are well-known human pathogens (e.g. *Staphylococcus aureus*), cutaneous species of *Staphylococcus* play multiple roles in maintaining skin health [6,11]. Species within this genus can also participate in ancient processes like malodour production. We previously identified a monophyletic group of coagulase-negative staphylococci (CoNS) including *Staphylococcus hominis* and *Staphylococcus lugdunensis*, which are able to metabolize the human apocrine gland-derived metabolite, Cys-Gly-3M3SH, and convert it to an odorous molecule we associate with human malodour [3,12,14]. Interestingly, different isolates of the same species of *Staphylococcus* can have varying levels of activity against Cys-Gly-3M3SH, suggesting as yet undiscovered genetic features that could control malodour formation [12,15].

Studies understanding the diversity within microbiomes have traditionally involved amplicon (e.g. 16S rRNA) sequencing of mixed communities or cultured isolates [16,18]. Next-generation sequencing (e.g. Illumina) then unlocked the ability to perform metagenomics enabling deep sequencing of these microbiome communities without the bias introduced by amplicon sequencing [19]. The development of high-accuracy long-read sequencing technologies (e.g. Oxford Nanopore) further equipped metagenomic studies with the ability to generate longer sequences and hence could provide more complete genes, operons and even more complete genomes [20]. In this study, we combine the strengths of long-read sequencing and large-scale isolate culturing methods to characterize a microbiome community. We isolated the axillary staphylococci sampled from a single individual to understand the different genetic components that form the *Staphylococcus* community in a distinct body site. We adapted a low-cost high-throughput DNA extraction and whole-genome sequencing pipeline to enable the genetic assessment of these isolates. Through this, we reveal the genetic differences among these *Staphylococcus* isolates which could contribute to maintaining the skin microbiome community and ultimately skin health. This collection of staphylococci contributes to the establishment of the York Skin Microbiome (YSM) isolate resource of genetically diverse isolates from a variety of genera from matched individuals.

## Methods

### Sampling procedures

The adult male volunteer was recruited for axillary swabbing at Unilever R&D, Bebington, UK. The volunteer was in good general health, had not used any cosmetic products for 24 h before the study, was not taking antibiotics, had no active skin conditions and has not suffered from eczema in the last 5 years nor have ever had psoriasis. Axillary swabs were collected by swabbing in a linear motion 20 times using eSwabs (Copan) on both underarms and then placed into Amies transport medium.

### Bacterial culturing

Swabs were plated onto SS solid media (10 g l^−1^ tryptone, 5 g l^−1^ Lemco powder, 3 g l^−1^ yeast extract, 13 g l^−1^ agar no. 1, 10 g l^−1^ sodium pyruvate, 0.5 g l^−1^ glycine, 22.5 g l^−1^ potassium thiocyanate, 1.2 g l^−1^ disodium hydrogen orthophosphate dihydrate, 0.67 g l^−1^ sodium dihydrogen phosphate, 2 g l^−1^ lithium chloride, 10 ml l^−1^ glycerol and pH 7.2) or ACP solid media (39.5 g l^−1^ Blood Agar base no. 2, 3 g l^−1^ yeast extract, 2 g l^−1^ glucose, 5 ml l^−1^ Tween 80, 50 ml l^−1^ defibrinated horse blood and 100 mg l^−1^ fosfomycin) to enrich for staphylococci and corynebacteria, respectively. Colonies were allowed to grow for up to 2 days at 37 °C. Colonies were picked and grown overnight in brain-heart infusion broth+1% Tween-80 (BHIT) at 37 °C, shaking at 200 r.p.m. Cultures were stocked in BHIT supplemented with 10% glycerol in 2 ml 96-well plates.

### DNA extraction

Total (genomic and plasmid) DNA extraction was performed using a previously reported procedure [21] with the following changes: the enzymatic lysis buffer used also included 125 µg ml^−1^ lysostaphin, the enzymatic lysis step was performed over 5 h and DNA precipitation was performed for 15 min at room temperature.

### DNA library preparation, whole-genome sequencing and assembly

The modified Oxford Nanopore Native Barcoding Kit 96 V14 DNA library preparation protocol reported in Herman *et al*. [21] was used. The prepared library was loaded into a PromethION R10.4.1 flow cell, and sequencing was performed for 72 h and base-called using the singleplex high-accuracy model, 400 bps on MinKNOW 23.07.5. The reads were assembled with the EPI2ME Labs wf-bacterial-genomes isolate workflow [Flye [22], Medaka (Oxford Nanopore Technologies) and Prokka [23]].

### Bioinformatic tools

Multilocus sequence typing (MLST) phylogeny analyses were performed on RStudio 2024.01.1 by first calculating the pairwise mismatches (distances) between the allelic profiles of all known sequence types of *Staphylococcus epidermidis* and *S. hominis* available on PubMLST (www.pubmlst.org, accessed on 29 August 2025) using the dplyr package. The distances were then used to construct neighbour-joining trees using the ape package.

Pairwise genome comparisons of all assembled genomes were performed using the ANIm [24] analysis on pyANI v0.2.12 [25]. The genomes were dereplicated with drep v3.4.2 [26] using Mash [27] and MUMmer 3.0 [28], with a primary clustering average nucleotide identity (ANI) cutoff of 95% to distinguish between different species and a secondary clustering ANI cutoff of 99.5% to capture strain diversity to derive a representative set of genomes. The core genomes were determined using Roary [29] with an 80% blastp cutoff. PhyML 3.3 [30] trees of the core genome alignments of the representative genomes were constructed using the HKY85 substitution model with approximate likelihood ratio tests. Trees were visualized on Geneious Prime 2024.0.7 (Biomatters). 16S rRNA alignments were performed using the Clustal Omega algorithm and visualized on Geneious Prime 2024.0.7 (Biomatters).

Pangenome analysis was performed using the anvi-pan-genome package and visualized on anvi’o v8 [31]. Relevant gene clusters were extracted and functionally analysed with eggNOG-mapper v2 [32,33] to determine the respective COG categories. Metabolic pathways were identified using blastKOALA and KEGG Reconstruct [34,35]. Phage defence systems were predicted using PADLOC v4.3 [36].

## Results

### Isolation and genetic characterization of axillary *Staphylococcus*

**Fig. 1.**
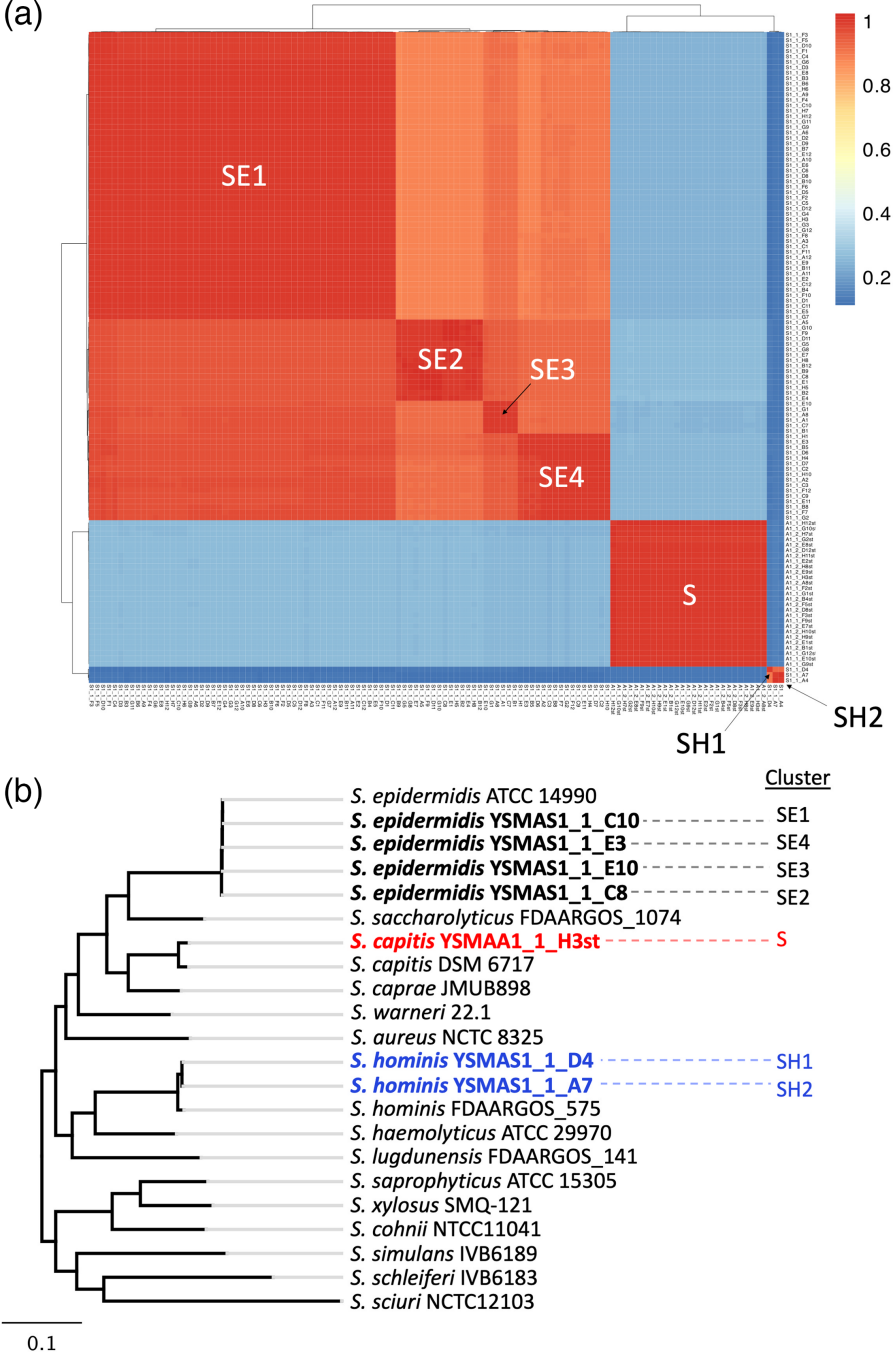
Diversity of 120 axillary *Staphylococcus* isolates. (**a**) A Hadamard matrix was constructed using the product of the pairwise average nucleotide identities and the pairwise alignment coverage and plotted using the pheatmap package on R. Isolate clusters were identified as SE1-4, SH1-2 or S. (**b**) Core genome maximum-likelihood tree of all seven dereplicated isolates with type strains of known cutaneous staphylococci. Clusters from (**a**) were identified next to the respective isolates and colour coded by species.

The axilla of the volunteer was swabbed and then the captured material was plated onto a selective agar to enrich for cutaneous staphylococci. Ninety-six isolates were harvested and stocked in the York Skin Microbiome (YSM) collection. The genomic DNA from these isolates was then extracted for long-read whole-genome sequencing using long-read sequencing on the PromethION platform. We generated complete genome sequences together with accompanying plasmid sequences for 93 of the 96 isolates. Using MLST [37], 90 of these isolates were determined to be *S. epidermidis* and 3 were *S. hominis* (Tables S1 and S2, available in the online Supplementary Material). To determine the diversity of these isolates, we generated a Hadamard matrix (Fig. 1a) using the pairwise average nucleotide identities, and the coverage of each alignment was calculated on pyani [38]. The 90 * S*. *epidermidis* isolates are grouped into 4 distinct clusters (SE1, SE2, SE3 and SE4) with isolates of SE1 dominating this population of *S. epidermidis* isolates. The three *S*. *hominis* isolates were found to be from two separate clusters (SH1 and SH2). We then performed species-specific phylogenetic analyses of the sequence types identified within our *Staphylococcus* isolates against all known MLST allelic profiles of the respective species. For *S. epidermidis*, clusters SE1 and SE4 were most similar, followed by SE2. However, cluster SE3 was found to be considerably more distant (Fig. S1). The MLST phylogenetic analysis of the *S. hominis* isolates suggested that SH1 and SH2, while clearly genetically distant, share a more recent common ancestor than most other sequence types within this species (Fig. S2).

We also unexpectedly identified 27 additional isolates of staphylococci using corynebacteria-enriching media containing fosfomycin, which we will include in our downstream analyses [21]. Analysis using pyani suggested that these 27 isolates are genetically highly similar and hence are grouped in the same cluster (S) (Fig. 1a). Unsurprisingly, data from Fig. 1(a) suggest that we have highly similar genomes in the library of *Staphylococcus* isolates from the volunteer due to indiscriminate large-scale isolation. Hence, we performed dereplication of the isolates using drep [26] with a secondary cluster threshold of 99.5% to define a set of seven genomes to represent isolates from clusters SE1, SE2, SE3, SE4, SH1, SH2 and S for use in downstream analysis. The core genome of the seven dereplicated isolates and type strains of other skin-associated staphylococci was derived and aligned using Roary [39] (Tables S3–S6). The resulting alignment was then used to derive a maximum-likelihood tree (Fig. 1b). The core genome of isolate YSMAA1_1_H3st, representing isolate cluster S (Fig. 1a, Table S7), clustered with *Staphylococcus capitis* and hence was assigned the species. The full 16S sequences of YSMAA1_1_H3st and *S. capitis* DSM 6717 were found to be identical, further confirming the species assignment (Fig. S3). This approach provided us with complete genomes of multiple genetically different staphylococci (four *S*. *epidermidis*, two *S*. *hominis* and one *S*. *capitis*) from the same individual which enables us to understand how these bacteria may have interacted in the microbiome they were isolated from.

### Species- and isolate-specific genetic traits reveal potential roles and functional networks within a single environment

**Fig. 2.**
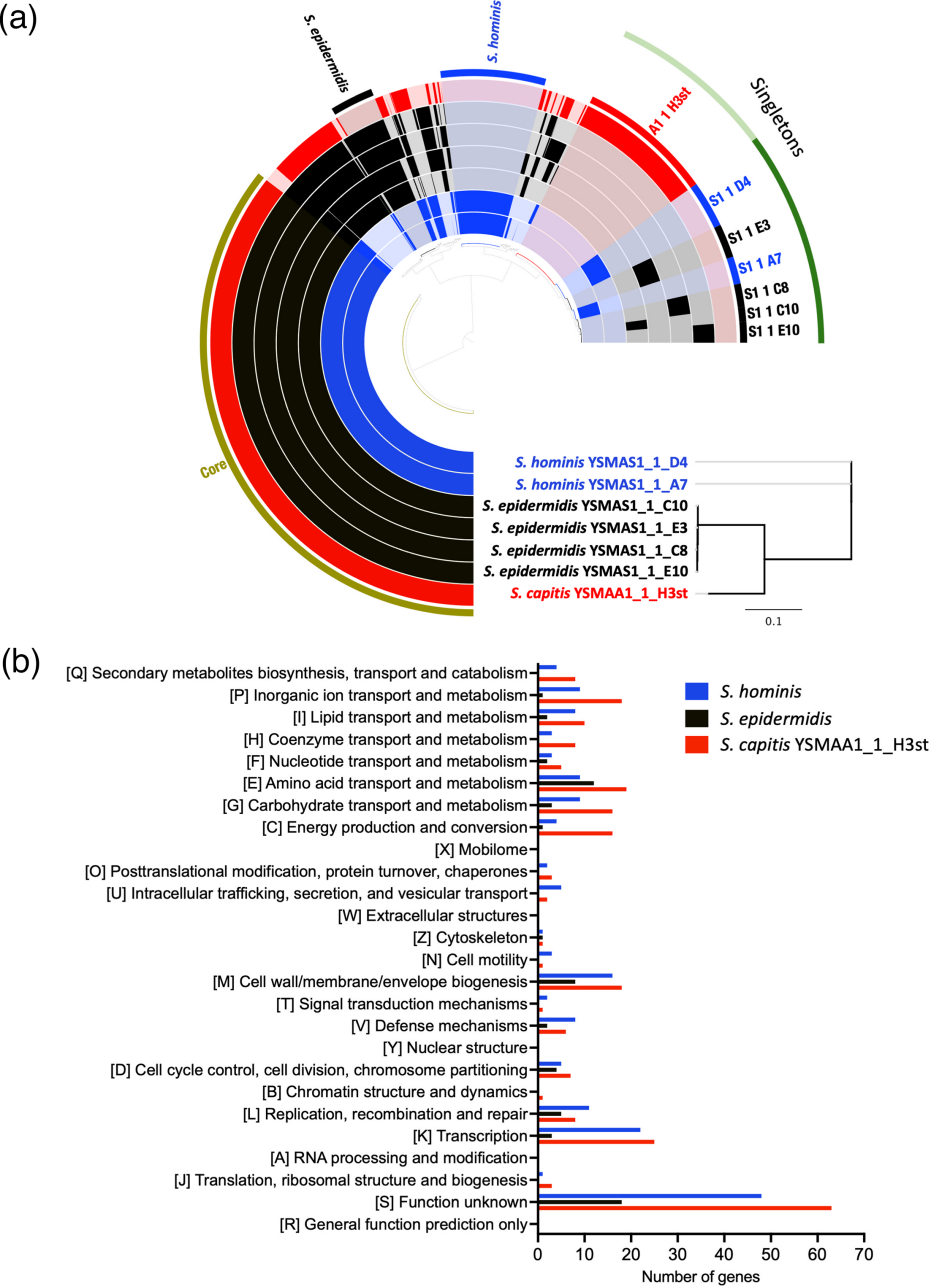
(a) Pangenome analysis of the seven dereplicated axillary Staphylococcus isolates identified species-specific clusters (*S. epidermidis* and * S. hominis*) and gene clusters belonging to only one isolate (singletons). The A1 1 H3st gene cluster identified in *S. capitis* YSMAA1_1_H3st was used in species-specific comparisons. The pangenome was calculated and visualized on anvio-8. (b) COG categories were assigned to species-specific gene clusters using eggNOG-mapper and visualized on GraphPad Prism 10.

We then performed a pangenome analysis using anvi’o [31] (Fig. 2a) which allowed us to identify genes specific to each species as well as genes only found in individual isolates (singletons) with the aim of revealing the roles of each genetically distinct isolate in this environment.

The pangenome analysis identified 1,609 core genes shared by all tested isolates of this genus with an additional 116 core * S. epidermidis* genes and 281 core *S. hominis* genes. These genes were assigned putative functions using eggNOG-mapper to map to clusters of orthologues genes (COGs), [32,33] to help elucidate the roles of the various species in the underarm of this single individual. As we were only able to isolate one subtype of *S. capitis*, we included the singletons identified for isolate *S. capitis* YSMAA1_1_H3st to represent the species. Major species-level differences were in the number of genes predicted to be involved in nucleotide related processes like transcription (K) and replication, recombination and repair (L) and also cell surface biogenesis (M) (Fig. 2b) . We also observed significant differences in the number of genes predicted to be involved in transport processes and metabolism, suggesting that there may be species-specific pathways. For example, this analysis suggested the *S. epidermidis* exclusive presence of the *argBCD* genes encoding proteins responsible for the conversion of *N*-acetylglutamate to *N*-acetylornithine during ornithine biosynthesis. In contrast, we found a complete metabolic pathway for the use of sialic acid as a carbon source in both *S. hominis* isolate types, which were not present in *S. epidermidis*. The genes resembled those found and characterized in *S. aureus*, in which study it was also noted that the same genes were absent in * S. epidermidis*, consistent with its inability to grow with Neu5Ac as the sole carbon source [40]. These data suggest at least one clear metabolic difference between *S. hominis* and *S. epidermidis* in addition to their difference in thioalcohol-based malodour production [3,14, 41].

**Fig. 3.**
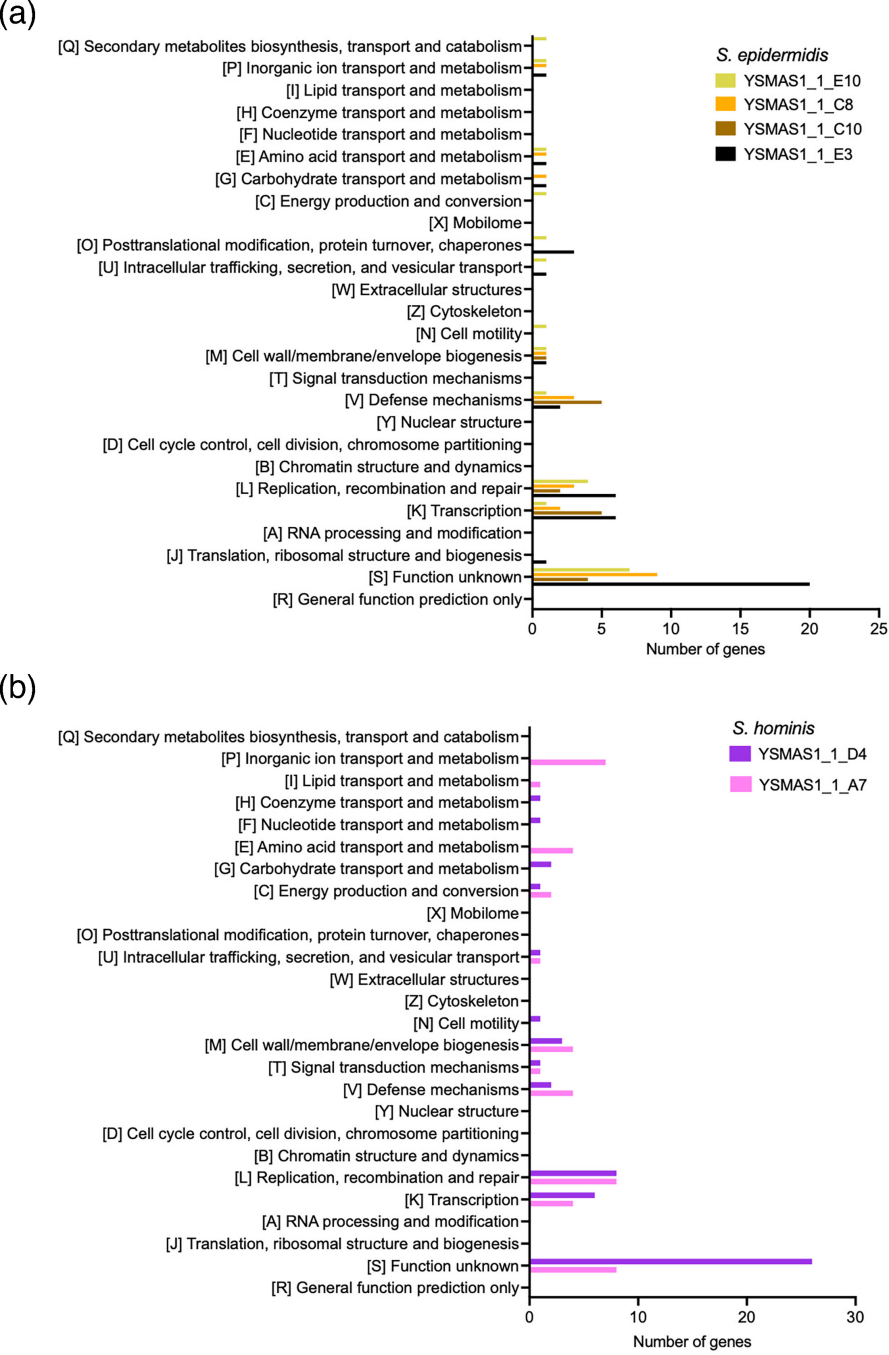
COG categories were assigned to singletons from (**a**) *S. epidermidis* and (**b**) *S. hominis* isolates using eggNOG-mapper and visualized on GraphPad Prism 10.

To further resolve the diversity of these isolates, we performed the same functional analysis on the singletons of all *S. hominis* and *S. epidermidis* isolates and compared them to other isolates of the same species (Fig. 3). Even within species-level comparisons, we observe the largest differences in transcription (K) and replication, recombination and repair (L).

This pangenome analysis workflow was also used to demonstrate the suitability of the isolates that were chosen to represent the isolate clusters reported in Fig. 1. Isolates within each cluster were compared with each other to identify non-core genes (i.e. not found in every isolate), and the representative isolates were assessed for the presence of these non-core genes (Tables S3–S7). We were not able to identify any non-core genes in the *S. hominis* isolates of this study. Non-core genes which were found in the majority of the *S. epidermidis* and *S. capitis* isolates of each cluster were also found in each of the representative isolates. An exception to this was in cluster SE2 where there were plasmid-located genes found within 8 out of 15 isolates, not including the representative isolate YSMAS1_1_C8 (Table S4). However, functional annotation was only successful for one of these genes, which was likely to be a plasmid replication protein and hence deemed not to be critical in the downstream analysis.

**Fig. 4.**
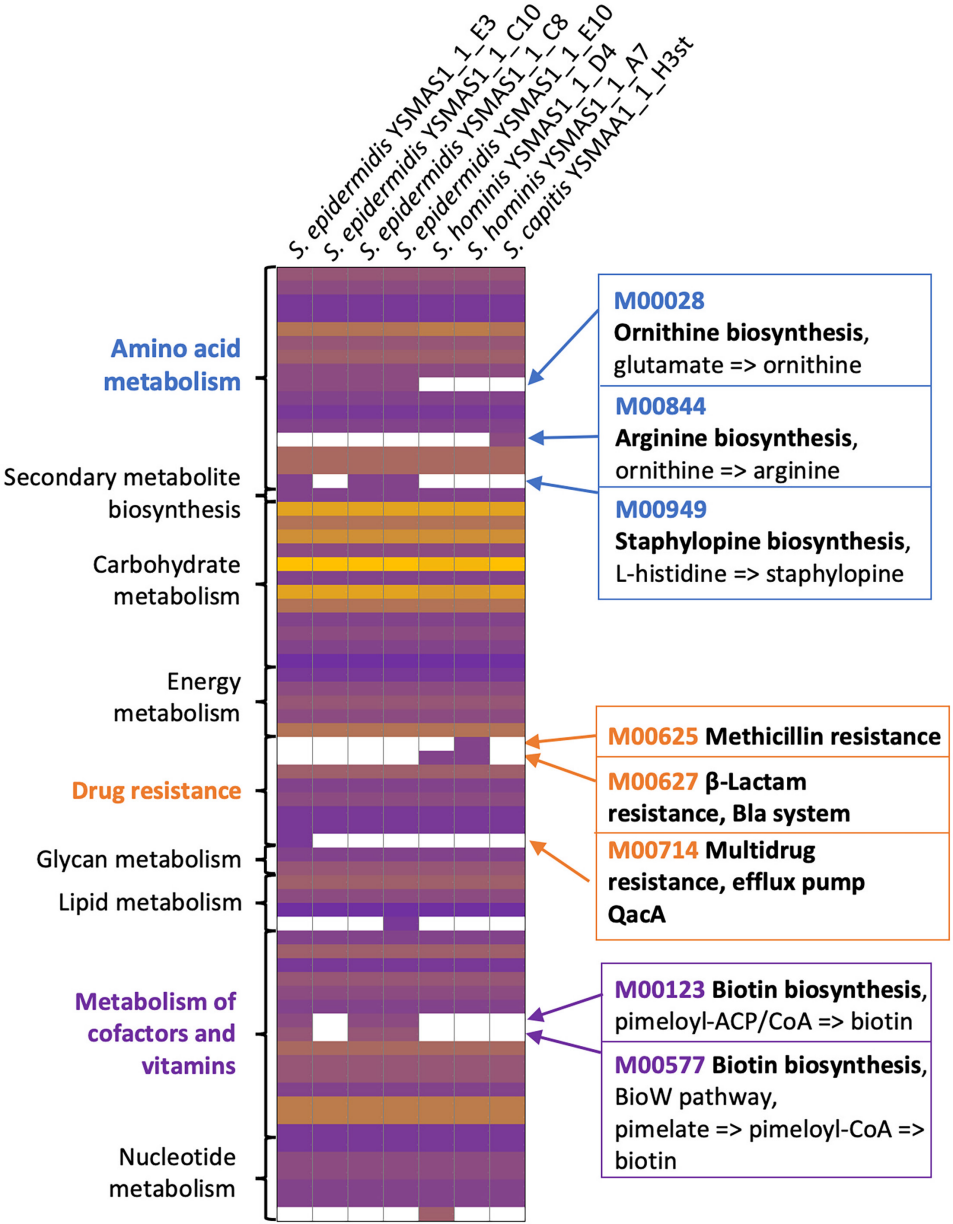
Metabolic pathways in genetically distinct axillary staphylococci reveal species and isolate-level variation within the isolates. Each complete KEGG module for each isolate was placed on a heatmap where the modules with the largest number of reactions are in yellow and the lowest in purple with absent modules in white. Modules of interest were described on the right of the heatmap. KEGG modules were grouped according to the categories on the left of the heatmap.

We also performed KEGG analysis on all seven isolates to map full pathways to complement the COG analysis, in particular to assess the presence of known metabolic pathways (Figs 4, S4 and S5). As observed in the COG analysis, there seems to be some correlation between the presence of some complete pathways and specific species. Here, we discuss some examples of the species- and strain-level differences which could allude to potential metabolic functions in the microbiome. Within the aa metabolism pathways, the ornithine biosynthesis module (M00028) seems to only be complete in *S. epidermidis* isolates and the entire module was found to be missing from *S. hominis* and *S. capitis*. This supports the COG analysis findings that identified the presence of *argBCD* only in *S. epidermidis*. Ornithine can be used in the biosynthesis of arginine as described in the KEGG module M00844 which is found to be present in all seven isolates (Fig. 4). For this purpose, ornithine could be imported from the environment using the ornithine transporter ArcD [42]. While the gene encoding for ArcD was identified in *S. capitis* YSMAA1_1_H3st, this transporter was not identified in the genomes of either of the two *S. hominis* isolates, suggesting the presence of a different ornithine transport pathway.

Similarly, the biotin biosynthesis pathways (M00123 and M00577) seem to only be present in *S. epidermidis* strains but are missing in *S. epidermidis* YSMAS1_1_C10, with the loss of the *bioDKFW* genes in a large strain-specific deletion on the chromosome (Fig. S6). The strain now resembles the *S. hominis* and *S. capitis* isolates in requiring exogenous biotin, which suggests that some subtypes of cutaneous *S. epidermidi*s could be the source of biotin for other auxotrophic species of this genus in the same community. The transporter BioY has been identified as a probable biotin uptake system in *S. aureus* [43] with the gene encoding BioY identified in all four isolates deficient in biotin biosynthetic pathways. Using the complete genomes of these isolates, we were able to perform an in-depth metabolic analysis to suggest the presence of metabolic cross-talk between members of the same skin microbiome.

We also observed differences within isolates of individual species. For example, the staphylopine biosynthesis pathway (M00949), deriving the zinc-binding siderophore from histidine [44], is found in three *S. epidermidis* isolates. The genes involved in staphylopine biosynthesis (*cntKLM*) are found in the same region of the chromosome as the biotin biosynthetic genes (*bioDKFW*) and hence the large chromosomal deletion also removed *cntKLM* from *S. epidermidis* YSMAS1_1_C10 (Fig. S6). Alongside * S. epidermidis* YSMAS1_1_C10, both *S. hominis* and the *S. capitis* isolates were also missing *cntKLM*. Rather than synthesizing staphylopine, these isolates could instead utilize and import this siderophore; however, they lack the direct orthologue of the * S. aureus* staphylopine CntABCDF ATP-binding cassette (ABC) permease [45] which is found downstream from *cntKLM* in all three *S. epidermidis* isolates with the complete staphylopine biosynthetic pathway.

In addition to metabolism, we also assessed the genomes for resistance cassettes and defence systems to understand the inherent protective measures which could maintain the community of staphylococci from the same site. In the KEGG analysis (Fig. 4), we observed the presence of modules providing resistance to antimicrobials including *β*-lactams via the bla system (M00627) and the mec systems (M00625) [46,47] in specific *S. hominis* isolates and to some antiseptics via the efflux pump QacA [48] in *S. epidermidis* YSMAS1_1_E3. These findings were confirmed using ResFinder (Table S8).

**Fig. 5.**
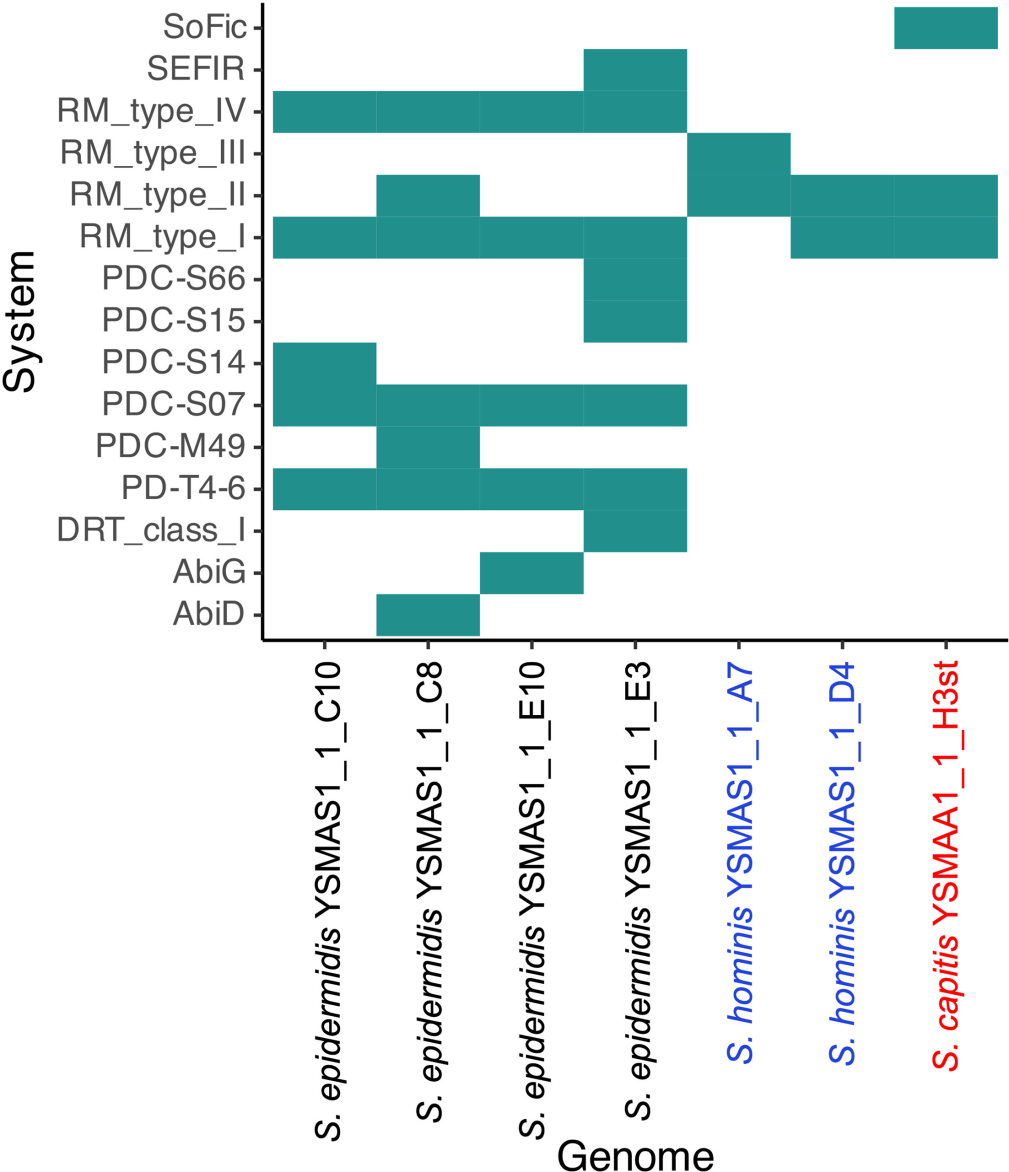
Phage defence systems were predicted in the seven dereplicated genomes using padloc. Only one of each system was identified in each relevant genome. Names of isolates were coloured according to the species they belong to (*S. epidermidis*, black; *S. hominis*, blue; and *S. capitis*, red). Data was visualized using R.

The COG analysis (Fig. 3) also identified species- and isolate-specific anti-phage defence systems (X) which suggest that we can expect an array of different systems in these isolates. We then analysed the genomes of these isolates on padloc [36] to reveal putative defence systems (Fig. 5). As expected, the padloc analysis predicted the presence of a wide range of defence systems. Some systems are found in all isolates of the same species like the type I and IV restriction modification (RM) systems in S. *epidermidis* isolates and the type II RM system in *S. hominis* isolates. However, as suggested by the species-specific COG analyses (Fig. 3), we also see isolate-specific systems. Notably, in *S. epidermidis*, we observed the presence of the type II RM system, the type I defence-associated reverse transcriptase (DRT_class_I), the abortive infection-related systems (AbiG and AbiD) and SEFIR in either of the four dereplicated isolates. Further, we also identified putative defence systems including some previously reported by Vassallo *et al*. [49], but these were not found in both *S. hominis* isolates and *S. capitis* YSMAA1_1_H3st. In *S. hominis*, predicted defence systems were limited to RM systems with the isolates either possessing a type III or a type I RM system alongside the type II RM system. We could use information from this analysis to aid in the design of genetic tools to target specific species or even specific isolates which will be invaluable in the understanding of the communities of the skin microbiome.

## Discussion

Recent advances in sequencing technologies have played a crucial role in revolutionizing our understanding of the human skin microbiome. Both short- and long-read sequencing technologies have provided us with the means to unlock the microbiome to study the abundance of different species in a single community through metagenomics and also enable cheaper whole-genome sequencing of isolates. In this study, we have proposed the use of a *Staphylococcus*-focused isolation approach involving the use of a low-cost total DNA extraction procedure and whole-genome sequencing for the isolation and characterization of cutaneous species. We tested this approach using isolates derived from a single volunteer which allowed us to assemble a library of 120 staphylococcal isolates containing 7 genetically distinct lineages of isolates from 3 different species.

The distribution of these seven lineages, having *S. epidermidis* as the most common, followed by *S. hominis* and then one isolate of another CoNS, is consistent with that observed in other species-level analyses of the skin and axilla microbiome [50,54]. However, the power of these new data enables the resolution of these communities to the strain level, which enabled us to identify genetic differences between isolates of the same species in the same niche, such as those for the biosynthesis of ornithine, staphylopine and biotin (Figs 4, S4 and S5). While this needs experimental verification, it does suggest that one lineage of *S. epidermidis*, which also happens to be that cultured most frequently, is one that appears to be a ‘cheater’ in an environment where other bacteria in the same niche produce biotin that it could scavenge.

The identification of four distinct lineages of *S. epidermidis* from a single individual’s axilla also raises interesting questions about the spatial organization of these bacteria on the skin. Little is known about whether distinct lineages are living separately in the same area together, or as a mixed population. The four lineages are different enough to have come from separate colonization events, but the dynamics of these processes are not understood. There is evidence of movement of skin *S. epidermidis* strains across human skin sites [55], and although this study did not include the axilla, there is no *a priori* reason not to assume that axilla isolates could not have originated from other skin sites. Temporal data would provide great insight into whether these four lineages are stably maintained or transient. Another study examining the skin microbiome across time does suggest there is retention at the subspecies level, which was informed using single nucleotide polymorphisms (SNP) data from shotgun metagenomics to identify subspecies of *S. epidermidis* and demonstrate their temporal stability [56]. The ability to now compare routinely fully closed genomes over time, should provide additional information about the evolution of strains within the community, including horizontal gene transfer (HGT) as well as SNP variation.

While the strength of this study is the strain-level resolution and capturing of the strains for downstream biological analysis, our results may be influenced by limitations in current isolation methods. Our protocols yielded a disproportionately large number of *S. epidermidis* isolates, potentially underrepresenting other *Staphylococcus* species. To address this, future studies could explore the design of targeted enrichment media aimed at improving species diversity. Insights from the KEGG analysis (Fig. 4) could inform the development of such media, demonstrating the practical utility of this genomic dataset beyond descriptive analysis.

Our workflow, powered by Oxford Nanopore’s long-read whole-genome sequencing technology, provided us with the ability to genetically characterize the library of isolates at a low per-sample cost and could potentially be adapted to other laboratory setups. The methods and findings of this study provide a platform to establish larger-scale studies of volunteers and isolates of their skin microbiome, not just on healthy individuals but could also be adapted for studies on diseased skin. The strain-level resolution described by our approach provides unparalleled access to fine-scale genetic variation, offering a powerful tool for dissecting microbe–microbe and microbe–host interactions. Ultimately, such insights will be critical for understanding how specific microbial strains contribute to skin health and disease and for identifying potential targets for therapeutic modulation of the skin microbiome.

## Supplementary material

10.1099/mgen.0.001531 Uncited Supplementary Material 1.

## Abbreviations

BHIT: brain-heart infusion broth+1% Tween-80
CoNS: coagulase-negative staphylococci
MLST: multilocus sequence typing
RM: restriction modification
