# Supplementary material for: High-resolution Staphylococcus profiling reveals intra-species diversity in a single skin niche
Source: Microb Genom. 2025 Oct 8;11(10):001531. doi: 10.1099/mgen.0.001531 (PMC12507337; doi:10.1099/mgen.0.001531)
Supplement: Uncited Supplementary Material 1. [file mgen-11-01531-s001.pdf]

Supplementary Data:

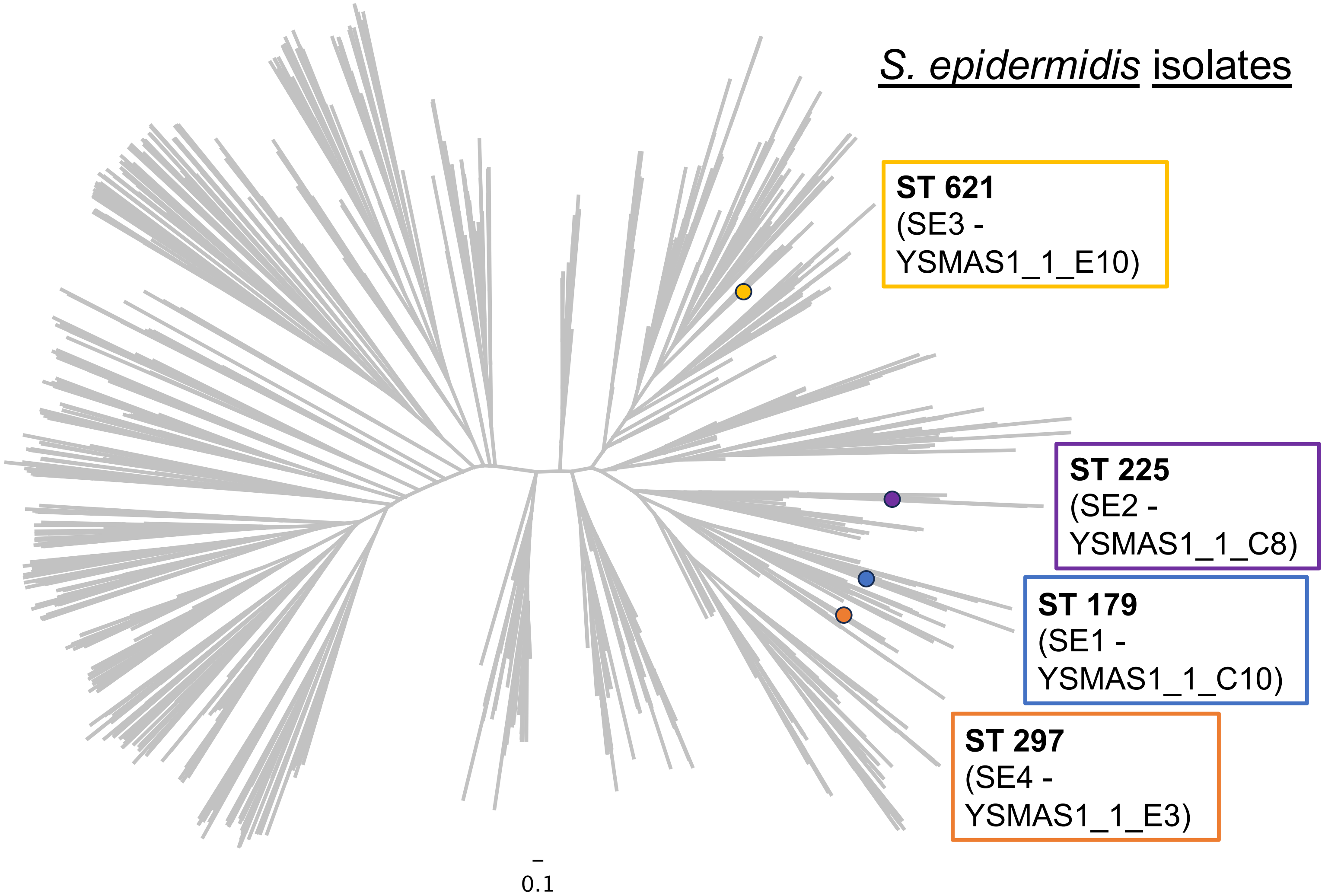

Supplementary Fig 1. Phylogenetic analysis of *S. epidermidis* MLST sequence types using the defined allelic profiles on PubMLST (www.pubmlst.org). The four clusters (SE1-4) were identified by the sequence types and the representative isolate.

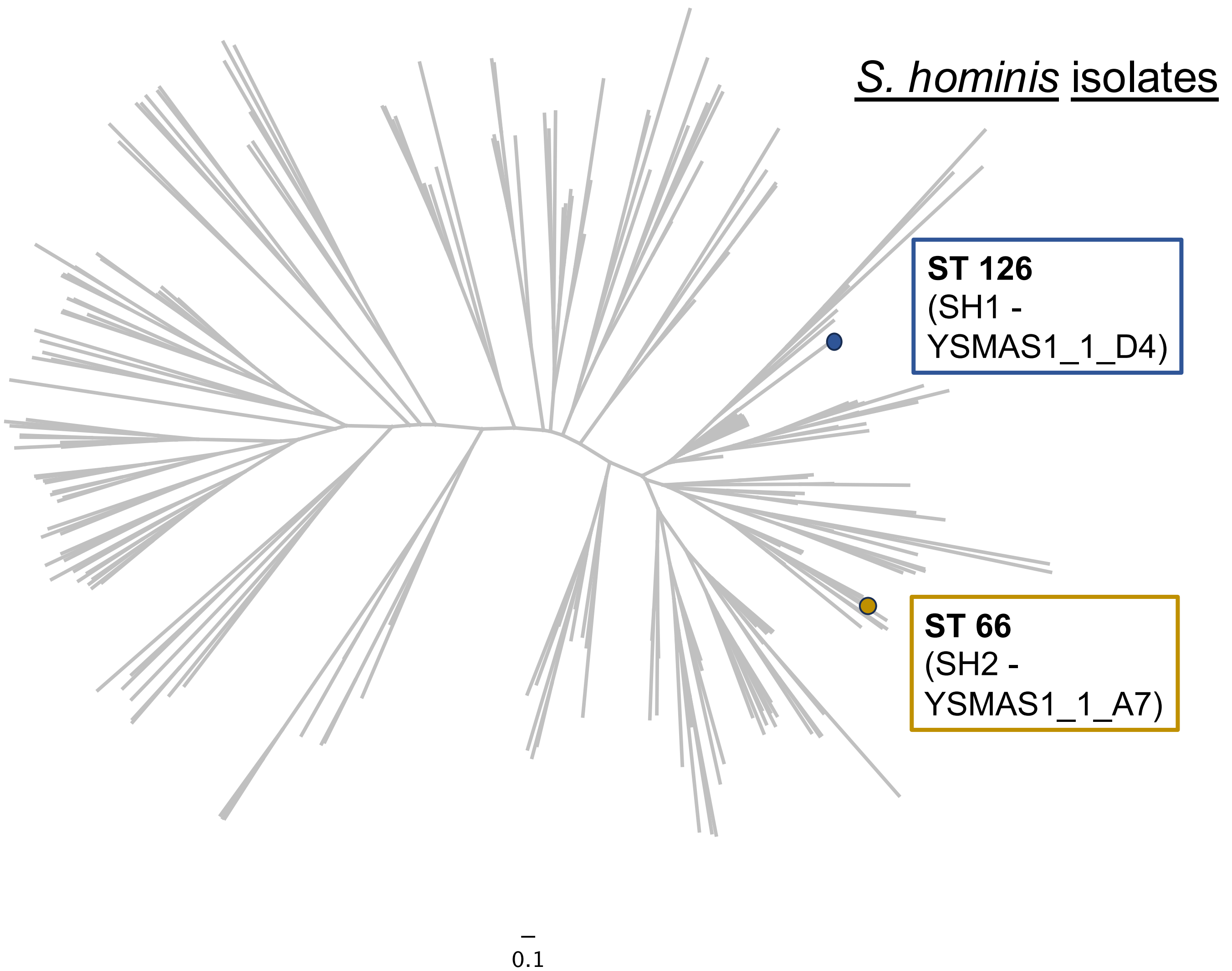

Supplementary Fig 2. Phylogenetic analysis of *S. hominis* MLST sequence types using the defined allelic profiles on PubMLST (www.pubmlst.org). The two clusters (SH1-2) were identified by the sequence types and the representative isolate.

```
Consensus                                    TATGGAGAGTTTGATCCTGGCTCAGGATGAACGCTGGCGGCGTGCCTAATACATGCAAGTCGAGCGAACAGACGAGGAGCTTGCTCCTCTGACGTTAGCGGCGGACGGGTGAGTAACACG   120
A1_1_H3st - 16S rRNA                         ........................................................................................................................   120
Staphylococcus_capitis_DSM_6717 - 16S rRNA   ........................................................................................................................   120

Consensus                                    TGGATAACCTACCTATAAGACTGGGATAACTTCGGGAAACCGGAGCTAATACCGGATAACATGTTGAACCGCATGGTTCAACAGTGAAAGACGGTCTTGCTGTCACTTATAGATGGATCC   240
A1_1_H3st - 16S rRNA                         ........................................................................................................................   240
Staphylococcus_capitis_DSM_6717 - 16S rRNA   ........................................................................................................................   240

Consensus                                    GCGCCGCATTAGCTAGTTGGTAAGGTAACGGCTTACCAAGGCAACGATGCGTAGCCGACCTGAGAGGGTGATCGGCCACACTGGAACTGAGACACGGTCCAGACTCCTACGGGAGGCAGC   360
A1_1_H3st - 16S rRNA                         ........................................................................................................................   360
Staphylococcus_capitis_DSM_6717 - 16S rRNA   ........................................................................................................................   360

Consensus                                    AGTAGGGAATCTTCCGCAATGGGCGAAAGCCTGACGGAGCAACGCCGCGTGAGTGAAGAAGGTCTTCGGATCGTAAAACTCTGTTATTAGGGAAGAACAAATGTGTAAGTAACTATGCAC   480
A1_1_H3st - 16S rRNA                         ........................................................................................................................   480
Staphylococcus_capitis_DSM_6717 - 16S rRNA   ........................................................................................................................   480

Consensus                                    GTCTTGACGGTACCTAATCAGAAAGCCACGGCTAACTACGTGCCAGCAGCCGCGGTAATACGTAGGTGGCAAGCGTTATCCGGAATTATTGGGCGTAAAGCGCGCGTAGGCGGTTTTTTA   600
A1_1_H3st - 16S rRNA                         ........................................................................................................................   600
Staphylococcus_capitis_DSM_6717 - 16S rRNA   ........................................................................................................................   600

Consensus                                    AGTCTGATGTGAAAGCCCACGGCTCAACCGTGGAGGGTCATTGGAAACTGGAAAACTTGAGTGCAGAAGAGGAAAGTGGAATTCCATGTGTAGCGGTGAAATGCGCAGAGATATGGAGGA   720
A1_1_H3st - 16S rRNA                         ........................................................................................................................   720
Staphylococcus_capitis_DSM_6717 - 16S rRNA   ........................................................................................................................   720

Consensus                                    ACACCAGTGGCGAAGGCGACTTTCTGGTCTGTAACTGACGCTGATGTGCGAAAGCGTGGGGATCAAACAGGATTAGATACCCTGGTAGTCCACGCCGTAAACGATGAGTGCTAAGTGTTA   840
A1_1_H3st - 16S rRNA                         ........................................................................................................................   840
Staphylococcus_capitis_DSM_6717 - 16S rRNA   ........................................................................................................................   840

Consensus                                    GGGGGTTTCCGCCCCTTAGTGCTGCAGCTAACGCATTAAGCACTCCGCCTGGGGAGTACGACCGCAAGGTTGAAACTCAAAGGAATTGACGGGGACCCGCACAAGCGGTGGAGCATGTGG   960
A1_1_H3st - 16S rRNA                         ........................................................................................................................   960
Staphylococcus_capitis_DSM_6717 - 16S rRNA   ........................................................................................................................   960

Consensus                                    TTTAATTCGAAGCAACGCGAAGAACCTTACCAAATCTTGACATCCTCTGACCCCTCTAGAGATAGAGTTTTCCCCTTCGGGGGACAGAGTGACAGGTGGTGCATGGTTGTCGTCAGCTCG  1080
A1_1_H3st - 16S rRNA                         ........................................................................................................................  1080
Staphylococcus_capitis_DSM_6717 - 16S rRNA   ........................................................................................................................  1080

Consensus                                    TGTCGTGAGATGTTGGGTTAAGTCCCGCAACGAGCGCAACCCTTAAGCTTAGTTGCCATCATTAAGTTGGGCACTCTAAGTTGACTGCCGGTGACAAACCGGAGGAAGGTGGGGATGACG  1200
A1_1_H3st - 16S rRNA                         ........................................................................................................................  1200
Staphylococcus_capitis_DSM_6717 - 16S rRNA   ........................................................................................................................  1200

Consensus                                    TCAAATCATCATGCCCCTTATGATTTGGGCTACACACGTGCTACAATGGACAATACAAAGGGTAGCGAAACCGCGAGGTCAAGCAAATCCCATAAAGTTGTTCTCAGTTCGGATTGTAGT  1320
A1_1_H3st - 16S rRNA                         ........................................................................................................................  1320
Staphylococcus_capitis_DSM_6717 - 16S rRNA   ........................................................................................................................  1320

Consensus                                    CTGCAACTCGACTACATGAAGCTGGAATCGCTAGTAATCGTAGATCAGCATGCTACGGTGAATACGTTCCCGGGTCTTGTACACACCGCCCGTCACACCACGAGAGTTTGTAACACCCGA  1440
A1_1_H3st - 16S rRNA                         ........................................................................................................................  1440
Staphylococcus_capitis_DSM_6717 - 16S rRNA   ........................................................................................................................  1440

Consensus                                    AGCCGGTGGAGTAACCTTTTGGAGCTAGCCGTCGAAGGTGGGACAAATGATTGGGGTGAAGTCGTAACAAGGTAGCCGTATCGGAAGGTGCGGCTGGATCACCTCCTT              1548
A1_1_H3st - 16S rRNA                         ............................................................................................................              1548
Staphylococcus_capitis_DSM_6717 - 16S rRNA   ............................................................................................................              1548
```

Supplementary Fig 3. Full 16S rRNA alignment of YSMAA1_1_H3st and *S. capitis* DSM 6717 (type strain).

Super Categories	Categories	Module	
Amino acid metabolism	Serine and threonine metabolism	M00018	Threonine biosynthesis, aspartate => homoserine => threonine
Amino acid metabolism	Serine and threonine metabolism	M00621	Glycine cleavage system
Amino acid metabolism	Serine and threonine metabolism	M00555	Betaine biosynthesis, choline => betaine
Amino acid metabolism	Cysteine and methionine metabolism	M00021	Cysteine biosynthesis, serine => cysteine
Amino acid metabolism	Cysteine and methionine metabolism	M00017	Methionine biosynthesis, aspartate => homoserine => methionine
Amino acid metabolism	Branched-chain amino acid metabolism	M00019	Valine/isoleucine biosynthesis, pyruvate => valine / 2-oxobutanoate => isoleucine
Amino acid metabolism	Branched-chain amino acid metabolism	M00570	Isoleucine biosynthesis, threonine => 2-oxobutanoate => isoleucine
Amino acid metabolism	Branched-chain amino acid metabolism	M00432	Leucine biosynthesis, 2-oxoisovalerate => 2-oxoisocaproate
Amino acid metabolism	Arginine and proline metabolism	M00028	Ornithine biosynthesis, glutamate => ornithine
Amino acid metabolism	Arginine and proline metabolism	M00844	Arginine biosynthesis, ornithine => arginine
Amino acid metabolism	Arginine and proline metabolism	M00970	Proline degradation, proline => glutamate
Amino acid metabolism	Arginine and proline metabolism	M00972	Proline metabolism
Amino acid metabolism	Histidine metabolism	M00045	Histidine degradation, histidine => N-formiminoglutamate => glutamate
Amino acid metabolism	Aromatic amino acid metabolism	M00022	Shikimate pathway, phosphoenolpyruvate + erythrose-4P => chorismate
Amino acid metabolism	Aromatic amino acid metabolism	M00023	Tryptophan biosynthesis, chorismate => tryptophan
Amino acid metabolism	Other amino acid metabolism	M00949	Staphylopine biosynthesis, L-histidine => staphylopine
Biosynthesis of terpenoids and polyketides	Terpenoid backbone biosynthesis	M00876	Staphyloferrin A biosynthesis, L-ornithine => staphyloferrin A
Carbohydrate metabolism	Central carbohydrate metabolism	M00001	Glycolysis (Embden-Meyerhof pathway), glucose => pyruvate
Carbohydrate metabolism	Central carbohydrate metabolism	M00002	Glycolysis, core module involving three-carbon compounds
Carbohydrate metabolism	Central carbohydrate metabolism	M00003	Gluconeogenesis, oxaloacetate => fructose-6P
Carbohydrate metabolism	Central carbohydrate metabolism	M00307	Pyruvate oxidation, pyruvate => acetyl-CoA
Carbohydrate metabolism	Central carbohydrate metabolism	M00009	Citrate cycle (TCA cycle, Krebs cycle)
Carbohydrate metabolism	Central carbohydrate metabolism	M00010	Citrate cycle, first carbon oxidation, oxaloacetate => 2-oxoglutarate
Carbohydrate metabolism	Central carbohydrate metabolism	M00011	Citrate cycle, second carbon oxidation, 2-oxoglutarate => oxaloacetate
Carbohydrate metabolism	Central carbohydrate metabolism	M00004	Pentose phosphate pathway (Pentose phosphate cycle)
Carbohydrate metabolism	Central carbohydrate metabolism	M00006	Pentose phosphate pathway, oxidative phase, glucose 6P => ribulose 5P
Carbohydrate metabolism	Central carbohydrate metabolism	M00007	Pentose phosphate pathway, non-oxidative phase, fructose 6P => ribose 5P
Carbohydrate metabolism	Central carbohydrate metabolism	M00580	Pentose phosphate pathway, archaea, fructose 6P => ribose 5P
Carbohydrate metabolism	Central carbohydrate metabolism	M00005	PRPP biosynthesis, ribose 5P => PRPP
Energy metabolism	Carbon fixation	M00579	Phosphate acetyltransferase-acetate kinase pathway, acetyl-CoA => acetate
Energy metabolism	Methane metabolism	M00345	Formaldehyde assimilation, ribulose monophosphate pathway
Energy metabolism	Sulfur metabolism	M00176	Assimilatory sulfate reduction, sulfate => H2S
Energy metabolism	ATP synthesis	M00416	Cytochrome aa3-600 menaquinol oxidase
Energy metabolism	ATP synthesis	M00157	F-type ATPase, prokaryotes and chloroplasts

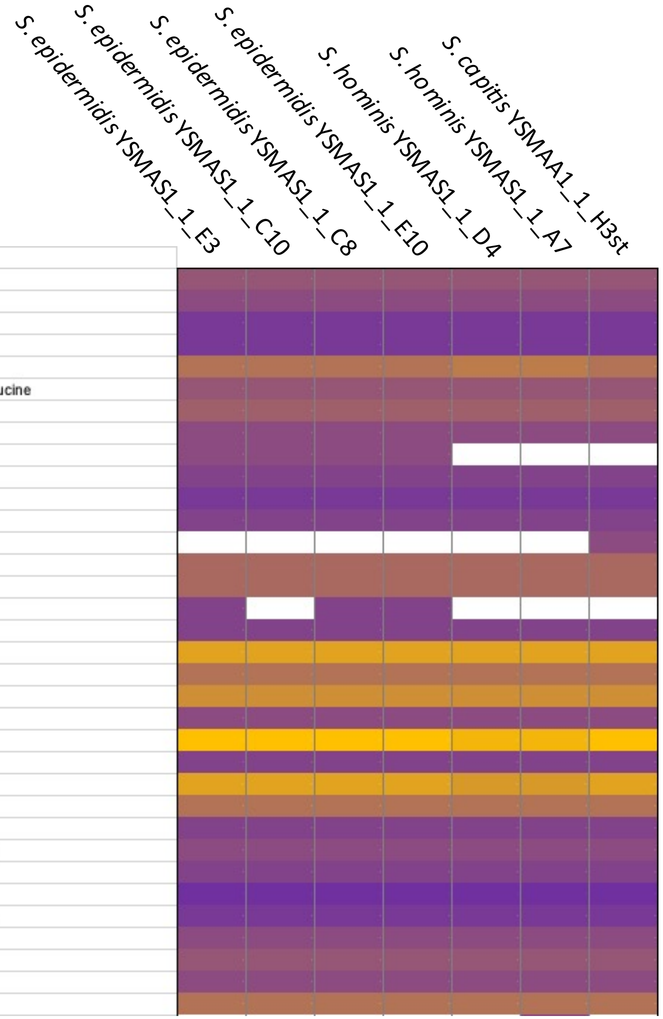

Supplementary Fig 4. KEGG modules shown in Fig 4 are identified. Included are modules from the Super Categories amino acid metabolism, biosynthesis of terpenoids and polyketides, carbohydrate metabolism and energy metabolism.

Super Categories	Categories	Module	
Gene set	Drug resistance	M00625	Methicillin resistance
Gene set	Drug resistance	M00627	beta-Lactam resistance, Bla system
Gene set	Drug resistance	M00725	Cationic antimicrobial peptide (CAMP) resistance, dltABCD operon
Gene set	Drug resistance	M00726	Cationic antimicrobial peptide (CAMP) resistance, lysyl-phosphatidylglycerol (L-PG) synthase MprF
Gene set	Drug resistance	M00730	Cationic antimicrobial peptide (CAMP) resistance, VraFG transporter
Gene set	Drug resistance	M00700	Multidrug resistance, efflux pump AbcA
Gene set	Drug resistance	M00702	Multidrug resistance, efflux pump NorB
Gene set	Drug resistance	M00714	Multidrug resistance, efflux pump QacA
Glycan metabolism	Nucleotide sugar biosynthesis	M00549	Nucleotide sugar biosynthesis, glucose => UDP-glucose
Glycan metabolism	Nucleotide sugar biosynthesis	M00909	UDP-N-acetyl-D-glucosamine biosynthesis, prokaryotes, glucose => UDP-GlcNAc
Lipid metabolism	Fatty acid metabolism	M00082	Fatty acid biosynthesis, initiation
Lipid metabolism	Fatty acid metabolism	M00083	Fatty acid biosynthesis, elongation
Lipid metabolism	Fatty acid metabolism	M00086	beta-Oxidation, acyl-CoA synthesis
Lipid metabolism	Lipid metabolism	M00098	Acylglycerol degradation
Metabolism of cofactors and vitamins	Cofactor and vitamin metabolism	M00899	Thiamine salvage pathway, HMP/HET => TMP
Metabolism of cofactors and vitamins	Cofactor and vitamin metabolism	M00125	Riboflavin biosynthesis, plants and bacteria, GTP => riboflavin/FMN/FAD
Metabolism of cofactors and vitamins	Cofactor and vitamin metabolism	M00916	Pyridoxal-P biosynthesis, R5P + glyceraldehyde-3P + glutamine => pyridoxal-P
Metabolism of cofactors and vitamins	Cofactor and vitamin metabolism	M00119	Pantothenate biosynthesis, valine/L-aspartate => pantothenate
Metabolism of cofactors and vitamins	Cofactor and vitamin metabolism	M00120	Coenzyme A biosynthesis, pantothenate => CoA
Metabolism of cofactors and vitamins	Cofactor and vitamin metabolism	M00883	Lipoic acid biosynthesis, animals and bacteria, octanoyl-ACP => dihydrolipoyl-H => dihydrolipoyl-E2
Metabolism of cofactors and vitamins	Cofactor and vitamin metabolism	M00123	Biotin biosynthesis, pimeloyl-ACP/CoA => biotin
Metabolism of cofactors and vitamins	Cofactor and vitamin metabolism	M00577	Biotin biosynthesis, BioW pathway, pimelate => pimeloyl-CoA => biotin
Metabolism of cofactors and vitamins	Cofactor and vitamin metabolism	M00126	Tetrahydrofolate biosynthesis, GTP => THF
Metabolism of cofactors and vitamins	Cofactor and vitamin metabolism	M00880	Molybdenum cofactor biosynthesis, GTP => molybdenum cofactor
Metabolism of cofactors and vitamins	Cofactor and vitamin metabolism	M00988	PreQ1 biosynthesis, GTP => 7-Aminomethyl-7-deazaguanine
Metabolism of cofactors and vitamins	Cofactor and vitamin metabolism	M00140	C1-unit interconversion, prokaryotes
Metabolism of cofactors and vitamins	Cofactor and vitamin metabolism	M00926	Heme biosynthesis, bacteria, glutamyl-tRNA => coproporphyrin III => heme
Metabolism of cofactors and vitamins	Cofactor and vitamin metabolism	M00116	Menaquinone biosynthesis, chorismate (+ polyprenyl-PP) => menaquinol
Metabolism of cofactors and vitamins	Cofactor and vitamin metabolism	M00364	C10-C20 isoprenoid biosynthesis, bacteria
Nucleotide metabolism	Purine metabolism	M00048	De novo purine biosynthesis, PRPP + glutamine => IMP
Nucleotide metabolism	Purine metabolism	M00049	Adenine ribonucleotide biosynthesis, IMP => ADP,ATP
Nucleotide metabolism	Purine metabolism	M00050	Guanine ribonucleotide biosynthesis, IMP => GDP,GTP
Nucleotide metabolism	Purine metabolism	M00053	Deoxyribonucleotide biosynthesis, ADP/GDP/CDP/UDP => dATP/dGTP/dCTP/dUTP
Nucleotide metabolism	Pyrimidine metabolism	M00052	Pyrimidine ribonucleotide biosynthesis, UMP => UDP/UTP,CDP/CTP
Nucleotide metabolism	Pyrimidine metabolism	M00938	Pyrimidine deoxyribonucleotide biosynthesis, UDP => dTTP

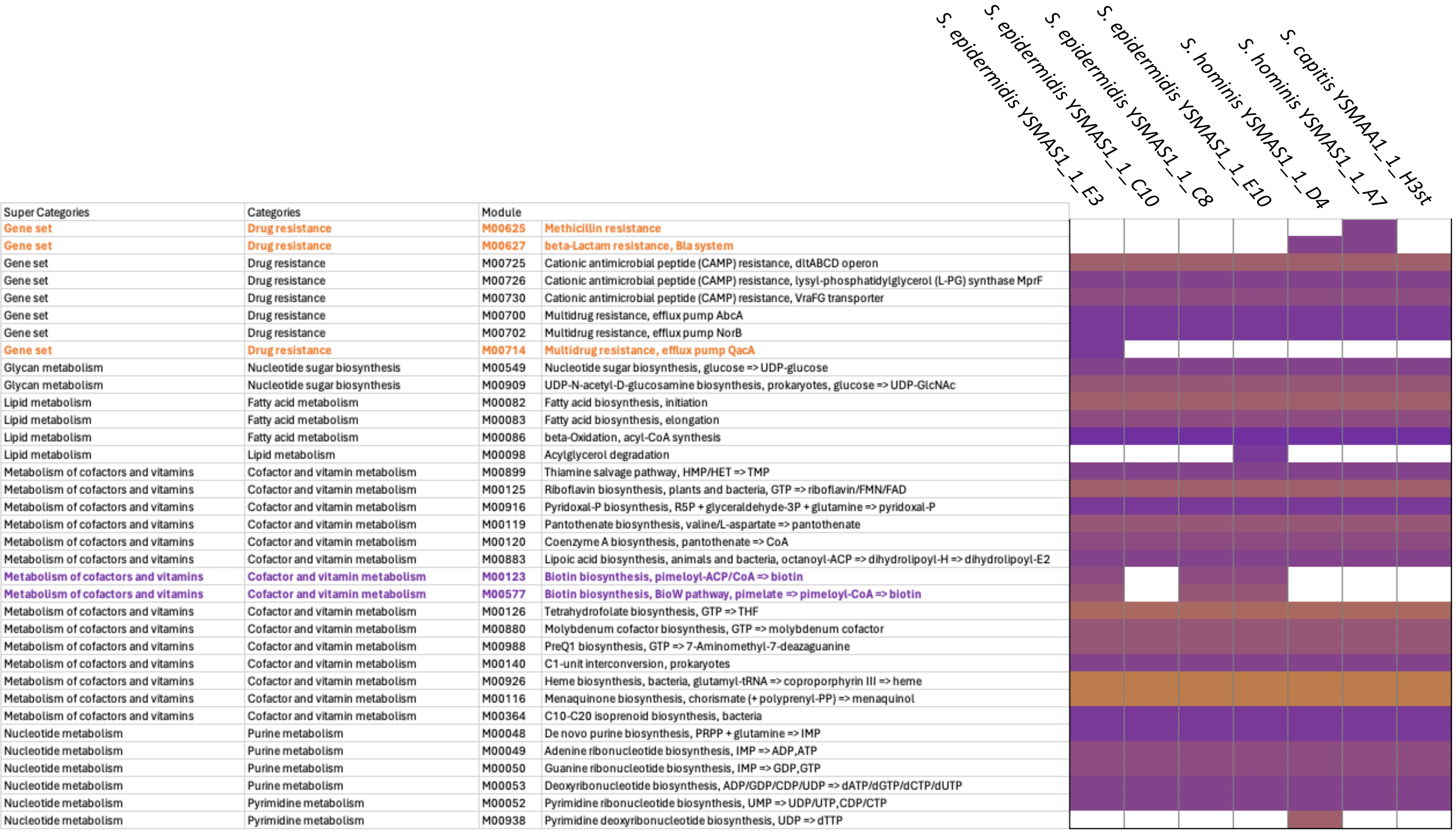

Supplementary Fig 5. KEGG modules shown in Fig 4 are identified. Included are modules from the Super Categories glycan metabolism, lipid metabolism, metabolism of cofactor and vitamins, nucleotide metabolism and the Category drug resistance.

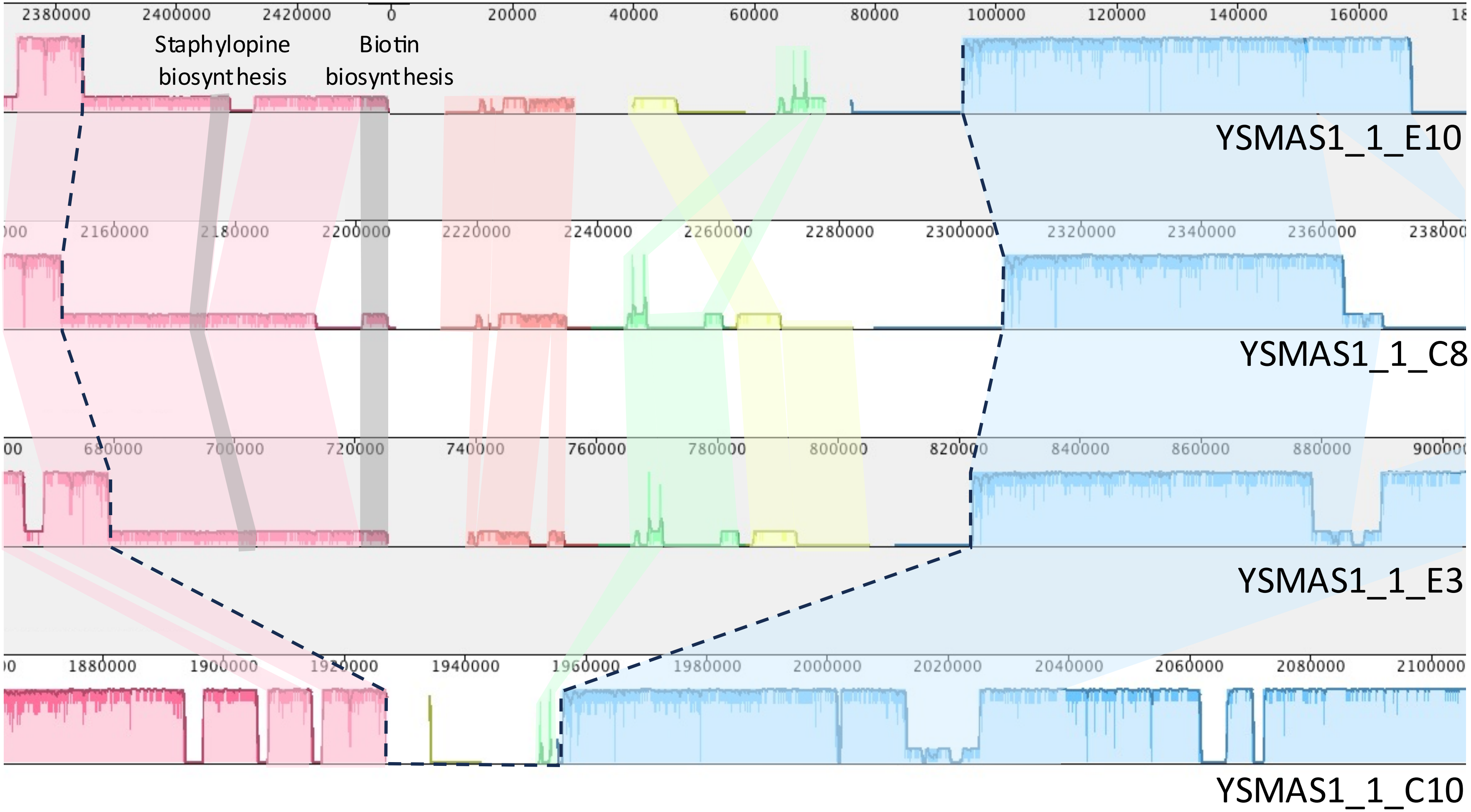

Supplementary Fig 6. *S. epidermidis* YSMAS1_1_C10 possesses a large chromosomal deletion which contains the staphylopine and biotin biosynthetic genes. The four representative genomes of S. epidermidis were aligned and visualised using Mauve (2015-02-25). The deletion was identified by the dashed lines. Similar genome sections were identified by coloured shadings.

Supplementary Table 1. Multilocus sequence typing (MLST) allelic profiles of representative *S. epidermidis* isolates. Listed for each isolate are the detected sequence type (ST), allele types of each locus used in the relevant MLST schemes (www.pubmlst.org).

S. epidermidis (scheme: sepidermidis)

Isolate	Cluster	ST	ArcC	AroE	Gtr	MutS	PyrR	TpiA	YqiL
YSMAS1_1_E3	SE4	297	1	2	2	2	2	1	3
YSMAS1_1_E10	SE3	621	7	1	1	6	2	1	1
YSMAS1_1_C8	SE2	225	1	13	7	2	2	1	29
YSMAS1_1_C10	SE1	179	1	2	2	2	1	1	1

Supplementary Table 2. Multilocus sequence typing (MLST) allelic profiles of representative *S. hominis* isolates. Listed for each isolate are the detected sequence type (ST), allele types of each locus used in the relevant MLST schemes (www.pubmlst.org).

S. hominis (scheme: shominis)

Isolate	Cluster	ST	ArcC	GlpK	Gtr	Pta	TpiA	Tuf
YSMAS1_1_A7	SH2	66	6	5	3	6	6	3
YSMAS1_1_D4	SH1	126	18	15	7	1	6	3

Supplementary Table 3: Non-core gene clusters from isolate cluster SE1 identified by the gene cluster number, location, COG category and description. The number of genomes within SE1 and whether or not the gene cluster is present in the representative genome S1_1_E10 were indicated.

Gene Cluster	Number of Genomes (Total = 53)	Present in S1_1_C10	Location	COG Category	Description
GC_00002060	52	Yes	Chromosome	S	Bifunctional serine threonine kinase and phosphorylase involved in the regulation of the pyruvate, phosphate dikinase (PPDK) by catalyzing its phosphorylation dephosphorylation
GC_00002061	52	Yes	Chromosome	O	Protein of unknown function (DUF2847)
GC_00002066	48	Yes	Chromosome	L	Belongs to the 'phage' integrase family
GC_00002069	48	Yes	Chromosome	S	D5 N terminal like
GC_00002070	48	Yes	Chromosome	K	Helix-turn-helix XRE-family like proteins
GC_00002072	48	Yes	Chromosome	-	-
GC_00002079	1	No	Plasmid	-	-
GC_00002082	1	No	Plasmid	L	Initiator Replication protein
GC_00002083	1	No	Plasmid	S	Possible lysine decarboxylase
GC_00002084	1	No	Plasmid	-	-
GC_00002086	1	No	Plasmid	-	-
GC_00002087	1	No	Plasmid	-	-
GC_00002088	1	No	Plasmid	-	-
GC_00002092	1	No	Plasmid	-	-
GC_00002093	1	No	Plasmid	-	-

Supplementary Table 4: Non-core gene clusters from isolate cluster SE2 identified by the gene cluster number, location, COG category and description. The number of genomes within SE2 and whether or not the gene cluster is present in the representative genome S1_1_C8 were indicated.

Gene Cluster	Number of Genomes (Total = 15)	Present in S1_1_C8	Location	COG Category	Description
GC_00002225	14	Yes	Chromosome	S	Phage portal protein, SPP1 Gp6-like
GC_00002227	8	No	Plasmid	-	-
GC_00002228	8	No	Plasmid	-	-
GC_00002229	8	No	Plasmid	L	Initiator Replication protein
GC_00002230	8	No	Plasmid	-	-
GC_00002231	8	No	Plasmid	-	-
GC_00002234	6	No	Plasmid	D	Plasmid recombination enzyme
GC_00002235	6	No	Plasmid	S	Protein of unknown function (DUF3139)
GC_00002237	6	No	Plasmid	K	Cro/C1-type HTH DNA-binding domain
GC_00002240	5	No	Plasmid	S	Possible lysine decarboxylase
GC_00002241	5	No	Plasmid	-	-
GC_00002243	5	No	Plasmid	-	-
GC_00002247	5	No	Plasmid	-	-
GC_00002248	3	No	Plasmid	L	Replication initiation factor
GC_00002251	1	No	Plasmid	N	Hep Hag repeat protein

Supplementary Table 5: Non-core gene clusters from isolate cluster SE3 identified by the gene cluster number, location, COG category and description. The number of genomes within SE3 and whether or not the gene cluster is present in the representative genome S1_1_E10 were indicated.

Gene Cluster	Number of Genomes (Total = 6)	Present in S1_1_E10	Location	COG Category	Description
GC_00002156	2	No	Plasmid	L	Initiator Replication protein
GC_00002160	2	No	Plasmid	-	-
GC_00002161	2	No	Plasmid	-	-
GC_00002162	2	No	Plasmid	-	-
GC_00002163	2	No	Plasmid	D	Plasmid recombination enzyme
GC_00002164	1	No	Plasmid	L	Replication initiation factor

Supplementary Table 6: Non-core gene clusters from isolate cluster SE4 identified by the gene cluster number, location, COG category and description. The number of genomes within SE4 and whether or not the gene cluster is present in the representative genome S1_1_E3 were indicated.

Gene Cluster	Number of Genomes (Total = 16)	Present in S1_1_E3	Location	COG Category	Description
GC_00002218	4	Yes	Chromosome	-	-
GC_00002219	4	Yes	Chromosome	T	Domain of unknown function (DUF4118)
GC_00002220	4	Yes	Chromosome	S	SEFIR domain
GC_00002222	4	Yes	Chromosome	-	-
GC_00002223	4	Yes	Chromosome	-	-
GC_00002224	4	Yes	Chromosome	T	catabolite gene activator and regulatory subunit of cAMP-dependent protein kinases
GC_00002225	4	Yes	Chromosome	E	Pyridoxal-dependent decarboxylase conserved domain
GC_00002227	4	Yes	Chromosome	P	ATP-driven potassium transport (or Kdp) system. This subunit is responsible for energy coupling to the transport system
GC_00002228	4	Yes	Chromosome	-	-
GC_00002229	4	Yes	Chromosome	-	-
GC_00002231	4	Yes	Chromosome	P	ATP-driven potassium transport (or Kdp) system. This subunit acts as a catalytic chaperone that increases the ATP- binding affinity of the ATP-hydrolyzing subunit KdpB by the formation of a transient KdpB KdpC ATP ternary complex
GC_00002232	4	Yes	Chromosome	L	RePlication protein
GC_00002233	4	Yes	Chromosome	KT	Transcriptional regulatory protein, C terminal
GC_00002234	4	Yes	Chromosome	V	ATPases associated with a variety of cellular activities
GC_00002236	4	Yes	Chromosome	P	ATP-driven potassium transport (or Kdp) system. This subunit binds and transports the potassium across the cytoplasmic membrane
GC_00002237	4	Yes	Chromosome	T	Histidine kinase-, DNA gyrase B-, and HSP90-like ATPase
GC_00002238	4	Yes	Chromosome	P	MFS/sugar transport protein
GC_00002241	4	Yes	Chromosome	P	P-type ATPase
GC_00002243	4	Yes	Chromosome	S	4Fe-4S single cluster domain
GC_00002244	4	Yes	Chromosome	K	Transcriptional regulatory protein, C terminal
GC_00002245	4	Yes	Chromosome	M	Protein of unknown function (DUF1541)
GC_00002247	1	No	Plasmid	-	-
GC_00002248	1	No	Plasmid	-	-
GC_00002249	1	No	Plasmid	-	-
GC_00002250	1	No	Plasmid	-	-
GC_00002252	1	No	Plasmid	D	Plasmid recombination enzyme

Supplementary Table 7: Non-core gene clusters from isolate cluster S identified by the gene cluster number, location, COG category and description. The number of genomes within S and whether or not the gene cluster is present in the representative genome A1_1_H3st were indicated.

Gene Cluster	Number of Genomes (Total = 27)	Present in A1_1_H3st	Location	COG Category	Description
GC_00000047	26	Yes	Chromosome	L	Replication initiation factor
GC_00000055	26	Yes	Plasmid	-	-
GC_00000074	26	Yes	Chromosome	M	C-terminus of bacterial fibrinogen-binding adhesin
GC_00002340	26	Yes	Chromosome	S	YoeB-like toxin of bacterial type II toxin-antitoxin system
GC_00002342	26	Yes	Chromosome	S	Domain of unknown function (DUF4870)
GC_00002344	26	Yes	Chromosome	S	Pfam:DUF867
GC_00002345	26	Yes	Chromosome	P	ABC transporter
GC_00002346	26	Yes	Chromosome	C	Catalyzes the reductive cleavage of azo bond in aromatic azo compounds to the corresponding amines. Requires NADH, but not NADPH, as an electron donor for its activity
GC_00002347	26	Yes	Chromosome	M	Bacterial capsule synthesis protein PGA_cap
GC_00002348	26	Yes	Chromosome	D	Antitoxin component of a toxin-antitoxin (TA) module
GC_00002351	26	Yes	Chromosome	K	Transcriptional regulator
GC_00002352	26	Yes	Chromosome	S	Poly-gamma-glutamate hydrolase
GC_00002353	26	Yes	Chromosome	M	Bacterial capsule synthesis protein PGA_cap
GC_00002354	26	Yes	Chromosome	GH	Belongs to the TPP enzyme family
GC_00002355	26	Yes	Chromosome	K	regulator
GC_00000066	19	Yes	Plasmid	U	Relaxase/Mobilisation nuclease domain
GC_00002360	17	Yes	Plasmid	S	Bacterial mobilisation protein (MobC)
GC_00002363	17	Yes	Plasmid	K	Cupin domain
GC_00002364	17	Yes	Plasmid	-	-
GC_00002365	17	Yes	Plasmid	-	-
GC_00002366	17	Yes	Plasmid	M	Acetyltransferase (GNAT) domain
GC_00002367	17	Yes	Plasmid	L	Replication protein
GC_00002368	17	Yes	Plasmid	L	Resolvase, N terminal domain
GC_00002370	17	Yes	Plasmid	S	Protein of unknown function, DUF536
GC_00002356	9	No	Plasmid	L	RePlication protein
GC_00002371	9	No	Plasmid	S	Staphylococcus haemolytic protein
GC_00002372	8	Yes	Plasmid	S	Bacterial mobilisation protein (MobC)

Supplementary Table 8. Antimicrobial resistance cassettes identified in all representative isolates using ResFinder.

Isolate	1	2	3	4
S. capitis YSMAA1_1_H3st	vga(A)LC, DQ823382, [lincomycin, clindamycin, dalfopristin, pristinamycin iia, virginiamycin m, tiamulin]			
S. hominis YSMAS1_1_A7	mecA, BX571856, [amoxicillin, ampicillin, cefepime, cefixime, cefotaxime, cefoxitin, ceftazidime, ertapenem, imipenem, meropenem, piperacillin]	blaZ, NZ_JVAT01000021, [amoxicillin, ampicillin, piperacillin, penicillin]	fusC, KF527883, [fusidic acid]	
S. hominis YSMAS1_1_D4	blaZ, CP003979, [amoxicillin, ampicillin, piperacillin, penicillin]			
S. epidermidis YSMAS1_1_C8	fosB, CP000029, [fosfomycin]			
S. epidermidis YSMAS1_1_C10	fosB, CP000029, [fosfomycin]	fusB, AY373761, [fusidic acid]		
S. epidermidis YSMAS1_1_E3	fosB, ACHE01000077, [fosfomycin]	fusC, KF527883, [fusidic acid]	msr(A), X52085, [erythromycin, azithromycin, telithromycin, quinupristin, pristinamycin ia, virginiamycin s]	mph(C), AF167161, [erythromycin, spiramycin, telithromycin]
S. epidermidis YSMAS1_1_E10	fosB, CP000029, [fosfomycin]	fusB, AY373761, [fusidic acid]		
